# Candidiasis in Pediatrics; Identification and *In vitro *Antifungal Susceptibility of the Clinical Isolates

**Published:** 2016-03-15

**Authors:** R Mohammadi, B Ataei

**Affiliations:** 1 **Department of Medical Parasitology and Mycology, School of Medicine, Isfahan University of Medical Sciences, Isfahan, Iran. **; 2 **Infectious Diseases and Tropical Medicine Research Center, Isfahan University of Medical Sciences, Isfahan, Iran. **

**Keywords:** Antifungal susceptibility, Candidemia, Pediatrics

## Abstract

**Background:**

Candida species are normal microflora of oral cavity, vagina, and gastrointestinal tract. They are the third most prevalent cause of pediatric health care–associated bloodstream fungal infection. This study aimed to provide an epidemiological feature of candidiasis and also presents an antifungal susceptibility profile of clinical *Candida* isolates among children.

**Materials and Methods:**

During July 2013 to February 2015, 105 patients from different hospitals of Isfahan, Iran, were examined for candidiasis by phenotypic tests. Samples were obtained from nail clippings, blood, thrush, BAL, urine, oropharynx, skin, and eye discharge. The age range of patients was between 18 days to 16 years. Genomic DNA of isolates was extracted and ITS1-5.8SrDNA-ITS2 region was amplified by ITS1 and ITS2 primers. The PCR products were digested using the restriction enzyme *Msp*I. Minimum inhibitory concentration (MICs) was determined using microdilution broth method according to the clinical and laboratory standards institute (CLSI) M27-A3 and M27-S4 documents.

**Results:**

Forty-three patients (40.9%) had *Candida* infection.The most clinical strains were isolated from nail infections (39.5%), and candidemia (13.9%). *Candida albicans* was the most prevalent species (46.5%). MICs ranges for amphotericin B, fluconazole, and itraconazole were (0.025-0.75 µg/ml), (0.125-16 µg/ml), and (0.094-2 µg/ml), respectively.

**Conclusion:**

Due to high incidence of *Candida* infections among children, increasing of fatal infection like candidemia, and emersion of antifungal resistance *Candida* isolates, early and precise identification of the *Candida* species and determination of antifungal susceptibility patterns of clinical isolates may lead to better management of the infection.

## Introduction

The prevalence of fungal infections has increased since the 1980s in different patient groups, particularly in young, immunosuppressed, and hospitalized patients, and connected to extra morbidity and mortality. Candida species are normal microflora of oral cavity, vagina, and gastrointestinal tract (1-3), and are responsible for different clinical forms of the infection, from mucocutaneous colonization to bloodstream fatal infections, for example Candida species are the third most prevalent cause of pediatric health care–associated bloodstream fungal infection in the United States and Europe (4). Candidosis results from an endogenous colonization; however, nosocomial transmission and resistant strains to antifungal agents propose new and remarkable problems (5). This investigation sets an epidemiologic study focusing on the etiologic agents of candidiasis in pediatrics and antifungal susceptibility pattern of Candida species due to various clinical forms of candidiasis among this population.

## Materials and Methods

**Isolates**

A total of 105 patients with suspected candidiasis were included in this cross-sectional study, from different hospitals of Isfahan, Iran, during July 2013 to February 2015. Specimens were collected from nail clippings, blood, thrush, BAL, urine, oropharynx, skin, eye discharge, and sore. All specimens were examined by direct microscopic examination with 15% potassium hydroxide (KOH), and culture on sabouraud glucose agar (Difco, Detroit, MI, USA), and CHROMagar Candida (Paris, France). 

**Molecular identification**

**DNA extraction**

The genomic DNA of all isolates was extracted according to the previously described phenol-chloroform method using boiling technique (6). Briefly, a piece of fresh and single colony was added to the 1.5 ml Eppendorf tube containing 300 μl of lysis buffer (200 mMTris-HCl (pH 7.5), 25 mM EDTA, 0.5% w/v SDS, 250 mMNaCl). The suspension was mixed with phenol chloroform, and centrifuged at 10,000 g for 10 min. DNA was precipitated with an equal volume of isopropanol and 0.1 volume of 3.0 M sodium acetate (pH 5.2) and the DNA pellet was washed with 70% ethanol, dried in air, suspended in 50 μl of distilled water and kept at -20°C.

**PCR-RFLP**

The ITS1-5.8SrDNA-ITS2 region was amplified using PCR mixture including 5μl of 10 × reaction buffer, 0.4 mM dNTPs, 1.5 mM MgCl2, 2.5 U of Taq polymerase, 30 pmol of both ITS1 (5′ -TCC GTA GGT GAA CCT GCG G-3′) and ITS4(5′ -TCC TCC GCT TAT TGA TAT GC-3′) primers (7) and 2μl of extracted DNA in a final volume of 50μl. During the second step, PCR products were digested with the restriction enzyme MspI (Fermentas, Vilnius, Lithuania). Five and 12μl of each PCR and RFLP products were separated by gel electrophoresis on 1.5 and 2% agarose gel (containing 0.5 μg/ml ethidium bromide), respectively.

In vitro antifungal susceptibility testing Minimum inhibitory concentration (MICs) was determined according to the recommendations stated in the clinical and laboratory standards institute (CLSI) M27-A3 and M27-S4 documents. Amphotericin B (AmB; Bristol-Myers-Squib, Woerden, The Netherlands), fluconazole (FLU; Pfizer Central Research, Sandwich, United Kingdom), and itraconazole (ITC; Janssen Research Foundation, Beerse, Belgium) were used for preparation of the CLSI microdilution trays. The antifungal agents were diluted in the standard RPMI-1640 medium (Sigma Chemical Co.) buffered to pH 7.0 with 0.165 M morpholinepropanesulfonic acid (MOPS) (Sigma) with L-glutamine without bicarbonate to yield two times concentrations and dispensed into 96-well microdilution trays at a final concentration of 0.016–16 μg/ml for AmB, ITC; and 0.063–64 μg/ml for FLU. All clinical isolates were cultured on malt extract agar (MEA, Difco, Detroit, MI, USA) at 35°C in dark and inoculum suspensions were prepared by harvesting the cell from 24 hours old cultures and were adjusted spectrophotometrically in saline to optical densities ranged 75-77% transmission. Final inoculum ranged from 2.5×10^3^ to 5×10^3^ CFU/ml as demonstrated by a quantitative colony count on Sabouraud’s dextrose agar (SDA, Difco, Detroit, MI, USA). MIC values were determined visually after 24h at 35°C. The resistance breakpoints are fluconazole ≥ 8, itraconazole ≥ 1.0, amphotericin B ≥ 1.0 (8, 9). 

## Results

**Figure1:**
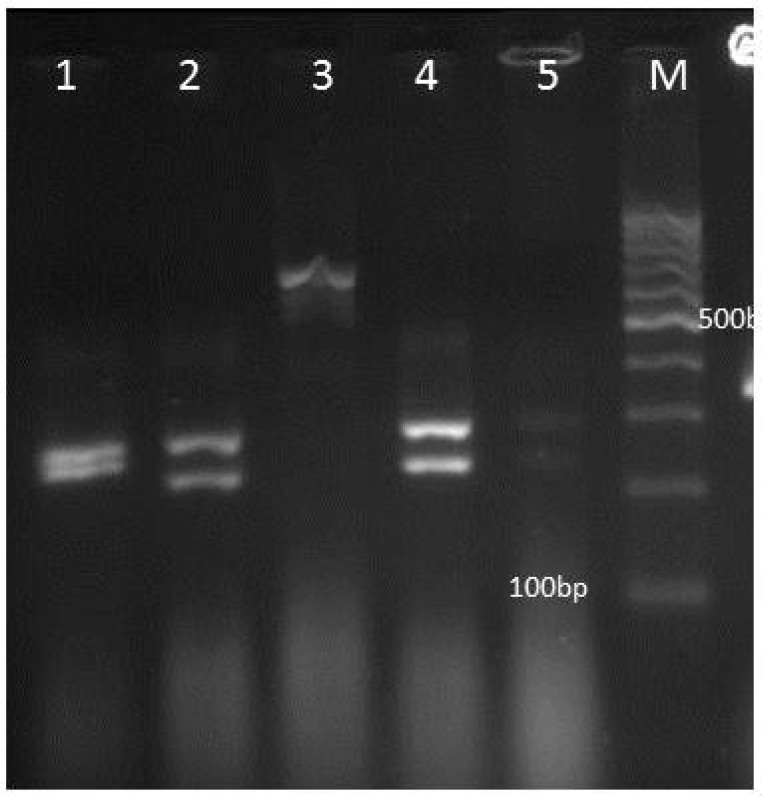
Agarose gel electrophoresis of ITS-PCR products of Candida isolates

Forty-three patients (40.9%) had Candida infection in the present study. The most samples were obtained from nail clippings (39.5%), blood (13.9%), and thrush (11.6%). Age range of patients was between 18 days to 16 years (mean age; 5.2 years). Male to female sex ratio was 24/19. One patient had chronic mucocutaneous candidiasis (CMC). He was underwent bone marrow transplantation. Candida albicans was the most common species isolated from patients (46.5%) followed by C. parapsilosis (18.6%) and C. kefyr (11.6%) (Figure1). Table I shows patients descriptions in details. MICs ranges for amphotericin B, fluc .0onazole, and itraconazole were (0.025-0.75 µg/ml), (0.125-16µg/ml), and (0.094-2 µg/ml), respectively (Table II). 

after digestion with MspII. Lanes 1:C.krusei, lanes 2,4,5: C. albicans, lane 3: C.

kefyr, and lanes M is 100 bp DNA size marker.

**Table I:** *Details of patients with candidiasis in the present study*

**No.**	**Sex**	**Age**	**Clinical location**	**Predisposing factors**	**Candida spp.**
**1**	M	5	Nail	Diabetes	*C. krusei*
**2**	M	6 mon	Thrush	-	*C. albicans*
**3**	M	9	BAL^*^	-	*C. albicans*
**4**	F	12	Nail	Diabetes	*C. krusei*
**5**	F	6	Nail	-	*C. parapsilosis*
**6**	M	8	Nail	Use of antibiotic	*C. parapsilosis*
**7**	F	6	Nail	Nutrients deficiency	*C. kefyr*
**8**	F	6	Thrush	-	*C. albicans*
**9**	F	14	Groin	-	*C. albicans*
**10**	F	8 mon	Thrush	-	*C. albicans*
**11**	F	16	Nail	Diabetes	*C. parapsilosis*
**12**	M	10	Nail	Leukemia	*C. parapsilosis*
**13**	F	7	Nail	Nutrients deficiency	*C. parapsilosis*
**14**	M	7 mon	Thrush	-	*C. albicans*
**15**	M	10	Oropharynx+BAL	Lymphoma	*C. albicans*
**16**	F	6	BAL	Use of antibiotic	*C. albicans*
**17**	M	1.5	Blood	Leukemia	*C. albicans*
**18**	F	6	Eye discharge	-	*C. albicans*
**19**	M	3	Sore	Burning	*C. parapsilosis*
**20**	F	1	Nail	-	*C. tropicalis*
**21**	F	1	Blood	-	*C. albicans*
**22**	M	3	Nail	Diabetes	*C. kefyr*
**23**	M	14	Oropharynx	Nutrients deficiency	*C. krusei*
**24**	F	6	Head	BM transplantation	*C. albicans*
**25**	M	6	Blood	Use of catheter	*C. albicans*
**26**	M	4	Nail	-	*C. albicans*
**27**	M	8	Oropharynx	-	*C. kefyr*
**28**	M	4	Skin	Use of antibiotic	*C. kefyr*
**29**	F	14	Blood	Leukemia	*C. albicans*
**30**	F	2	Nail	Lymphoma	*C. albicans*
**31**	F	3	Nail	-	*C. krusei*
**32**	F	3	Nail	-	*C. parapsilosis*
**33**	M	2	Urine	Use of antibiotic	*C. tropicalis*
**34**	M	10	Urine	Diabetes	*C. tropicalis*
**35**	M	1	Urine	-	*C. tropicalis*
**36**	M	2	Nail	-	*C. kefyr*
**37**	M	2	Nail	-	*C. parapsilosis*
**38**	M	4	Nail	-	*C. guilliermondii*
**39**	F	1.5	Groin	-	*C. albicans*
**40**	M	18 days	Blood	Use of catheter	*C. albicans*
**41**	F	35 days	Blood	Use of catheter	*C. albicans*
**42**	M	6.5	Skin	-	*C. glabrata*
**43**	M	55 days	Thrush	-	*C. albicans*

Mon: month, BAL: Broncho-alveolar lavage, BM: Bone marrow

**Table II:** *In vitro antifungal susceptibility testing of Candida spp.isolated from pediatrics*

**No.**	**Candida spp.** ** Candida spp.**	**AP MIC (µg/ml)**	**FL MIC (µg/ml)**	**IT MIC (µg/ml)**
**1**	*C. krusei*	0.5	1.5	0.5
**2**	*C. albicans*	0.025	1	1
**3**	*C. albicans*	0.5	1	0.125
**4**	*C. krusei*	0.094	1.5	1
**5**	*C. parapsilosis*	0.025	0.5	0.25
**6**	*C. parapsilosis*	0.5	0.5	0.5
**7**	*C. kefyr*	0.19	0.25	0.094
**8**	*C. albicans*	0.094	0.5	0.25
**9**	*C. albicans*	0.125	1	0.25
**10**	*C. albicans*	0.047	16	2
**11**	*C. parapsilosis*	0.5	0.125	0.5
**12**	*C. parapsilosis*	0.094	0.5	0.094
**13**	*C. parapsilosis*	0.5	0.125	0.5
**14**	*C. albicans*	0.19	0.75	0.25
**15**	*C. albicans*	0.094	0.5	0.5
**16**	*C. albicans*	0.025	0.5	0.094
**17**	*C. albicans*	0.094	0.25	0.94
**18**	*C. albicans*	0.19	0.125	0.5
**19**	*C. parapsilosis*	0.094	1	0.25
**20**	*C. tropicalis*	0.5	1	1
**21**	*C. albicans*	0.025	0.5	0.5
**22**	*C. kefyr*	0.125	0.25	0.094
**23**	*C. krusei*	0.19	4	1
**24**	*C. albicans*	0.125	0.125	0.094
**25**	*C. albicans*	0.5	0.5	0.25
**26**	*C. albicans*	0.19	0.25	0.125
**27**	*C. kefyr*	0.75	0.75	0.094
**28**	*C. kefyr*	0.094	0.25	0.94
**29**	*C. albicans*	0.5	1.5	0.094
**30**	*C. albicans*	0.19	0.5	0.5
**31**	*C. krusei*	0.025	1	1
**32**	*C. parapsilosis*	0.5	0.5	0.5
**33**	*C. tropicalis*	0.025	0.125	0.25
**34**	*C. tropicalis*	0.047	1	0.094
**35**	*C. tropicalis*	0.5	0.5	0.5
**36**	*C. kefyr*	0.094	1	0.5
**37**	*C. parapsilosis*	0.025	0.125	0.094
**38**	*C. guilliermondii*	0.125	0.75	0.5
**39**	*C. albicans*	0.19	0.25	0.5
**40**	*C. albicans*	0.094	0.5	0.25
**41**	*C. albicans*	0.19	0.125	0.25
**42**	*C. glabrata*	0.19	0.5	0.5
**43**	*C. albicans*	0.19	0.125	0.5

*AP: Amphotericin B, FL: Fluconazole, IT: Itraconazole.*

## Discussion

Several Candida species are colonized on the skin surfaces and mucosal layers of humans. Immunosuppressed patients are more susceptible to develop both superficial and life-threatening Candida infections (10). Candidiasis is also the most prevalent fungal infections in AIDS patients (10, 11). This group mainly infected to oropharyngeal candidiasis, which can lead to malnourishment and obstruct the absorption of medication. There was no any HIV+ patient, but 3 patients (7%) were diagnosed with oropharyngeal candidiasis and malnourishment. Although C. albicans is the most common species connected to candidiasis, the incidence of non-albicans Candida species is increasing. This alteration in epidemiology could be connected to prematurity, the severe illnesses or immunosuppression conditions, use of broad-spectrum antibiotics, and elderly (12). The prevalence of nail infections elevates with age, diabetes, nail trauma, circumferential circulation, long-time exposure to the pathogenic fungi, use of broad‑spectrum antibiotics, corticosteroid therapy, and immune system disorders (13). Candida nail infections occur in patients with chronic mucocutaneous candidiasis, and are found more frequently in females than males (14), in agreement with the present study. Nail infection affect the middle finger due to contact with Candida strains that are in the intestine or vagina (14, 15). Middle finger was affected in all patients with nail infection in the present study with the exception of 6 patients. Tortorano et al., in 2006, reported that in European countries, more than half of the candidaemia cases were caused by C. albicans, followed by C. glabrata (14%) C. parapsilosis (14%), C. tropicalis (7%), and C. krusei (2%) (16). Ajenjo et al. also revealed that the prevalence of C. albicans has altered in Chile, and an accelerating increase of non-albicans Candida infection has been noticed. They recognized C. parapsilosis as the most common species, followed by C. tropicalis, and C. glabrata. All isolates were susceptible to amphotericin B; however, 50% of the C. glabrata isolates were resistant to fluconazole (17), however, the etiologic agent of all candidemia cases in the present investigation was Candida albicans, and in vitro antifungal susceptibility pattern showed that all C. albicans isolated from bloodstream were susceptible to amphotericin B, and fluconazole. C. parapsilosis has appeared as a nosocomial fungal pathogen with clinical signs containing arthritis, endocarditis, endophthalmitis, peritonitis and fungaemia, usually connected to prosthetic devices or invasive procedures (18). In Spain, Canton et al., in 2011, showed that C. Parapsilosis is the second most frequently Candida spp. isolated from blood stream after C. albicans (18); whereas, no C. parapsilosis strain was isolated among candidemia cases in the present study. Candidaemia due to C. tropicalis has been connected to the malignancies, particularly in patients with neutropenia and leukemia (19), but in this investigation, the etiologic agent of candidemia in patients with leukemia (no. 17 and 29) was C. albicans. Candidaemia caused by C. glabrata has been described to be connected to the use of azoles like fluconazole (20). In the present investigation only one C. glabrata strain was isolated from skin lesions however, blood stream infection was not associated with C. glabrata. Candida guilliermondii was formerly unusual Candida spp., however, the prevalence of C. guilliermondii is increasing, too (20, 21). This investigation isolated a Candida guilliermondii (2.3%) from patient no. 38 with nail infection. Candida spp. causes candiduria in 22% of patients entered into the intensive care unit (ICU) (22), nevertheless 3 patients (7%) had candiduria in the present study. The colonization of Candida species in the respiratory tract is usual in the patients receiving mechanical ventilation for periods of longer than 2 days. This happens because of haematogenous spread or pulmonary aspiration of the substances of fungal colonies of oropharyngeal origin (23). A patient with oropharengeal candidosis was diagnosed in the present study (patient no. 15). Pathogenicity of Candida species depends on many virulence factors, such as biofilm formation, adherence ability to the host tissues and medical appliances like catheters, and secretion of some hydrolytic enzymes(24). Three out of 4 patients with candidemia used catheter as a predisposing factor of candidemia in the present investigation. It can connect to the biofilm formation on the catheters. Among the non-albicans Candida species, C. parapsilosis and C. tropicalis are usually susceptible to azoles; but, C. tropicalis is less susceptible to fluconazole than C. albicans (25, 26). It is in accordance with these findings because 2 out of 4 C. tropicalis were resistant to fluconazole. C. glabrata is intrinsically more resistant to antifungal drugs especially to fluconazole (21). C. glabrata strain (no. 42) was susceptible to all antifungal agents used in the present study. Seifi et al., (27) in 2013, reported 5.2% candiduria among children in Ahvaz, whereas 6.9% candiduric patients were diagnosed in the present study. In another study in Ilam (28), resistance rate of Candida strains isolated from children with oral candidosis and diaper dermatitis to fluconazole and itraconazole, was 43% and 34.2%, respectively, while 2.3% of clinical isolates were resistant to these antifungal drugs in the present study.

## Conclusion

Considering the high incidence of Candida infections caused by non-albicans species, increasing of fatal infection like candidemia, excessive exposure to the antifungal agents, and the appearance of antifungal resistance isolates, successful treatment of candidiasis is based on the early and precise identification of the Candida species and determination of antifungal susceptibility patterns of clinical isolates, as it was done in this study.
